# Unraveling the history of Tibetan Plateau growth inspires Earth system research

**DOI:** 10.1093/nsr/nwaf102

**Published:** 2025-03-21

**Authors:** Andreas Mulch

**Affiliations:** Senckenberg Biodiversity and Climate Research Centre, Germany; Institute of Geosciences, Goethe University Frankfurt, Germany

The snow-capped peaks and majestic heights of the world's highest places have impressed and inspired humankind throughout its history. Equally important, the Himalaya and Tibetan Plateau (TP) play a crucial role in a multitude of interactions that control the functioning and habitability of our planet: the southeastern plateau margin harbors hotspots of biodiversity [1], erosion and weathering affect Earth's climate through feedbacks with the global carbon cycle [2], and the TP reroutes atmospheric moisture and hence provides water for billions of people [3]. Tracking the growth history of the TP over geological time is a fundamental building block in elucidating some of these controls. Surface uplift occurs in response to geodynamic processes in the crust and mantle that are linked to Earth surface processes including climate-controlled erosion. Concurrently, climate elements such as atmospheric circulation, precipitation amount and seasonality as well as temperature are modulated by changes in Earth surface topography on a regional to global scale (e.g. [4–7]). Consequently, changes in these environmental conditions directly influence the biosphere from the diversity of communities to the structure of biomes [8].

This complex interplay among long-term changes in climate, topography and biodiversity has gained increased attention (e.g. [1,9,10]) and robust topography reconstructions can provide the basis for next-generation Earth system models. How do mountains grow? What are the underlying geodynamic mechanisms and how do high-elevation regions control geo-biosphere interactions? The key to answering the individual facets of these questions lies with the history of the Earth's surface.

In a paper published in this issue, Zhao *et al*. [11] analyzed combined surface elevation and climate histories of the early Cenozoic (∼54–44 Ma) basin deposits in eastern Tibet. They conclude that a westward sloping valley crossed Central Tibet all the way to its eastern termination with a progressively rising valley floor propagating to the west.

This diagnosis rests upon three pillars: (1) fossil plant assemblages document that the eastern strands of this Central Tibetan Valley transitioned from arid conditions between 54 and 46 Ma to a diverse, subtropical forest straddling the valley's major river system no later than 44 Ma. (2) Oxygen isotopic compositions (δ^18^O) of limestone carbonate from the fluvio-lacustrine deposits when combined with paleobotanical data point to a concurrent increase in surface elevation from ∼0.6 km to ∼3.0 km. This analysis exploits the observation that δ^18^O values of precipitation scale with elevation during ascent, cooling and condensation of humid air masses. δ^18^O values of fossil or sedimentary materials that record δ^18^O values of precipitation reflect the elevation at which deposition in the basin occurred. (3) When compared to Central Tibetan basins further to the west, the temporal and spatial pattern of surface rise of the valley floor renders it likely that stepwise east-to-west lithosphere removal and sedimentary fill of the valley topography conspired to compose a coherent, high-elevation southern TP region. It is easy to envisage that a rather complex middle Eocene Tibetan topography not only affected the strength of the (proto-)Asian monsoons but an extensive Central Tibetan Valley may have further served as a corridor for the longitudinal exchange and diversification of biota [12,13].

So how do studies of biotic and environmental change and the rise of mountains promote our predictive abilities of how the Earth system responds to disturbances? The stark elevation and climate gradients commonly encountered in topographically complex regions provide fertile ground for identifying thresholds to ecosystem-wide tipping. Tracking elevation and climate histories permits identification of rates and magnitudes of environmental change prior, during and after major disturbances. Recovering stable isotopic compositions of precipitation through time and space enhances our ability to modify and adapt global atmospheric circulation models to the effects of complex landscapes. Despite different forcings, the history of the TP witnessed Earth system-wide disturbances similar to those predicted for the future of Tibetan ecosystems and water resources. Paleoclimatic and biotic information that integrates the effects of mountain building and environmental change can hence become invaluable for refined predictions.

With majestic height comes majestic value. The breathtaking landscapes of the Himalaya and Tibetan Plateau not only remind us about the value of nature. Knowing that they harbor answers to some of the most fundamental questions in keeping the Earth habitable should be an inspiration for future scientific efforts.
